# An Investigation into the Pressures Experienced by Medical Masters Students during the COVID-19 Pandemic Based on the Perceived Stress Scale-14 and Its Alleviation Methods

**DOI:** 10.3390/healthcare10061072

**Published:** 2022-06-09

**Authors:** Heyu Meng, Jianjun Ruan, Yanqiu Chen, Zhaohan Yan, Xiangdong Li, Fanbo Meng

**Affiliations:** 1 Jilin Provincial Precision Medicine Key Laboratory for Cardiovascular Genetic Diagnosis, Jilin Provincial Cardiovascular Research Institute, Jilin University, Changchun 130033, China; hymeng20@mails.jlu.edu.cn (H.M.); ruanjj19@mails.jlu.edu.cn (J.R.); chenyq20@mails.jlu.edu.cn (Y.C.); yanzh20@mails.jlu.edu.cn (Z.Y.); lxd870112@jlu.edu.cn (X.L.); 2 Jilin Provincial Molecular Biology Research Center for Precision Medicine of Major Cardiovascular Disease, Jilin Provincial Cardiovascular Research Institute, Jilin University, Changchun 130033, China; 3 Jilin Provincial Engineering Laboratory for Endothelial Function and Genetic Diagnosis of Cardiovascular Disease, Jilin Provincial Cardiovascular Research Institute, Jilin University, Changchun 130033, China

**Keywords:** COVID-19, PSS-14, medical students, gender, relaxation method

## Abstract

The COVID-19 pandemic can be seen as a traumatic event during which time medical students have been required to perform dual roles both as students and as medical workers. In this study, we aimed to use the Perceived Stress Scale (PSS-14) to judge the pressures on medical students and to identify effective ways to relieve these pressures. In this cross-sectional study, the subjects were recruited under informed consent according to the Declaration of Helsinki. Students undertaking Masters degrees at the China-Japan Union Hospital of Jilin University were randomly selected and data were collected through questionnaire surveys. Our data showed significant differences in the levels of pressure experienced by Masters students (*p* < 0.05). In the student population that showed increased pressure, females were significantly more stressed than males (*p* < 0.05). In addition, the pressures persisted after a holiday period (*p* < 0.05) but were reduced by undertaking recreational activities. The psychological pressures resulting from the COVID-19 pandemic were higher in female medical students compared to males. We showed that recreational activities including chatting with friends reduced psychological pressures in female medical students.

## 1. Introduction

The current COVID-19 pandemic is a traumatic event that causes individuals to experience physical, emotional and psychological harm [1]. Strategies designed to prevent the spread of COVID-19 introduced new sources of life stress that have heavily disrupted the daily lives of people in China. Mental health is highly sensitive to traumatic events and can have major social and economic consequences [1]. The anxiety caused by COVID-19 is multifaceted and connected to different aspects of the population, society and health [2]. The teaching of medical students online limits physical interactions that are particularly important during learning. During the pandemic, students changed their learning methods and had to adapt to online teaching, which increased anxiety [3,4]. Before COVID-19, the risk of disease among medical students had an upward trend [5] as students are under pressure during their studies [6,7].

Recent studies from Chinese medical students found an increased prevalence of pressure during COVID-19 [8,9]. The pressures on young people have also increased during the COVID-19 pandemic [10,11]. In young people, work pressures are the biggest source of stress [12] and in particular, unemployment can be a major cause of stress. Due to societal changes, young people often face pressures associated with the need to study towards gaining further education and improving their quality of life. Also, young people often use their spare time to participate in various business training activities that can inadvertently increase stress and become additional sources of pressure.

Medical students undertaking Masters degrees are part of the young population who may experience pressures from several sources. Stress can be caused by pressures associated with obtaining employment after graduation as this is often a highly competitive process that requires major preparation before graduation [13]. To obtain better job opportunities and work environments, students are required to continue their studies during their careers often involving additional entrance examinations that result in added pressures [14]. In particular, medical degrees are longer than most other majors and necessitate the detailed learning of high volumes of medical knowledge [15].

In this study, the PSS-14 questionnaire was used to investigate the pressures experienced by medical students undertaking Masters Degrees in the China-Japan Union Hospital of Jilin University. To assess the impact of a holiday period, the survey was repeated after a three-day recreational break. We identified the main sources of stress in the group of medical students and discuss the main approaches that can be used to reduce stress in these individuals.

**Figure 1:**
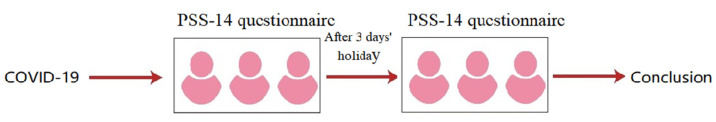
During the period of COVID-19 epidemic, two stress surveys were conducted for medical students. PSS-14 ques-tionnaire was used for the two surveys, with a 3-day vacation interval between the two questionnaires.

This study was designed to assess the stress levels in medical postgraduates during the COVID-19 pandemic using a questionnaire. The questionnaire was repeated after a three-day holiday to assess changes in stress level and explore the ways to reduce the stress. See Figure 1 for the flow chart.

## 2. Materials and Methods

### 2.1. Study Participants

This cross-sectional study was performed according to the declaration of Helsinki. All of the selected participants had their personal details removed and were anonymized to the investigators. Participants were randomly selected from medical masters students at the China-Japan Union Hospital of Jilin University and data were collected through a questionnaire survey. A total of 107 students participated in the survey. The exclusion criteria for this study were people who were familiar with the content of the test before the survey, people who have been previously diagnosed with depression or have received psychotherapy, patients with severe neurological diseases and people who were being treated for other diseases [16].

### 2.2. Study Methods

The questionnaire was distributed and accepted through a mobile app. All questions in the questionnaire are described in Chinese. The perforated Stress Scale-14 questionnaire was proposed by Cohen et al., in 1983 [17]. Measurements were made using the PSS-14 questionnaire that consisted of 14 questions with responses ranging from 0 to 4 based on the experiences and occurrences within the month prior to the survey [17]. The consistency of the PSS was 0.70 (Cronbach’s α coefficient) and the retest reliability was 0.73 over a short retest interval (several days). The way to analyze the questionnaire is to analyze the total score. The PSS-14 may have a score between 0 and 56, and 28 is the operating threshold [18]. A threshold stress score value of 28 was selected with students with perceived stress scores ≥28 identified as being more stressed, and students with perceived stress score <28 were identified as less stressed. The calculation formula is as follow. K is the number of test items, $\sum S_{i}^{2}$ is the score variation of all subjects on question i, $S_{x}^{2}$ is the variance of the total score of all subjects $\alpha =\frac{K}{K-1}\left(1-\frac{\sum \;S_{i}^{2}}{S_{x}^{2}}\right)$.

The subjects took two PSS-14 questionnaires. The first survey was conducted before a holiday and the second survey was conducted after the 3 day holiday. Both questionnaires consisted of 14 questions. Relaxation options were added to the second questionnaire and included details such as the time (mins) spent listening to music, exercising, watching movies and chatting with friends.

### 2.3. Statistical Analysis

SPSS24.0 (SPSS, Inc., Chicago, IL, USA) software was used for statistical analysis. The measured data were normally distributed and statistically described as the mean ± standard deviation. The differences between the groups were compared and analyzed using an independent *t*-test. The median and quartile intervals were used for statistical analysis and the differences between the groups were tested using a rank-sum test. *p* values < 0.05 were considered statistically significant.

### 2.4. Data Analysis

The stratification is based on the total score of the questionnaire. We conducted a hierarchical analysis of the data. The criteria for hierarchical analysis were the level of stress, gender and grade. After the three-day holiday, we carried out questionnaire statistics again and conducted hierarchical analysis. The criteria of hierarchical analysis were still the level of stress, gender and grade, but the statistics of relaxation mode was added. What we want to analyze is whether stress exists in medical students; whether the stress is related to gender and grade; whether stress exists after holidays and whether stress is related to gender; whether the holiday experience reduces the stress; the ways to relax during the holidays.

## 3. Results

### 3.1. Results of the First Questionnaire Survey (before the Holiday)

In the first questionnaire, 65 medical students completed the PSS-14 questionnaire and stress was commonly detected in the students (*p* < 0.05). The responses to Question 13 (Are you able to control the way you spend your time?) were believed to reflect the main cause of stress (*p* < 0.05) as shown in Table 1.

#### Stratification Analysis of Gender with Grades in the Increased Pressure Group Based on Results from the First Questionnaire Survey

Based on the results shown in Table 2, stratification analysis was conducted in the experimental group (≥28 scores) that included 40 females and 11 males. In the experimental group, according to the different admission times, 28 students were in the grade year 2018 and 20 students were in the grade year 2019. 3 students did not complete a grade on the questionnaire survey. Amongst the participants, female students were significantly more stressed than males (*p* < 0.05). The stratification analysis of grades was not statistically significant and the differences in grades were not associated with increased stress (*p* > 0.05).

### 3.2. Results of the Second (after the Holiday) Questionnaire Survey

A total of 42 medical students completed the second questionnaire following a three-day holiday. Our results showed that the pressures were still present in the same group of students. The results are presented in Table 3.

#### Stratification Analysis of Gender with Grades in the Increased Pressure Group from the Second Questionnaire Survey

Stratification analysis was conducted on the experimental group (≥28 scores) that consisted of 22 female and 5 male students. According to the different admission times, 8 students were in grade 2018 and 15 students weare in grade 2019, 4 students did not record a grade in the survey. Amongst the subjects, females were found to be more stressed than males (*p* < 0.05). The stratification analysis of grades was not statistically significant and the differences in grades was not associated with increased stress (*p* > 0.05) as shown in Table 4.

### 3.3. Analysis of the Increased Pressure Group before and after a Holiday

According to the data presented in Table 1 and Table 3, statistical analysis was performed on the increased pressure group before (*n* = 51) and after a holiday period (*n* = 27). The pressure values after the holiday were lower than those recorded before the holiday (pressure values were 30 and 31, respectively) (*p* > 0.05). The data are presented in Table 5.

#### Stratification Analysis of Gender in the Increased Pressure Group before and after a Holiday

Based on the data shown in Table 2 and Table 4, gender stratification was carried out for the increased pressure group before and after a holiday. It was found that the pressure values of the students decreased to varying degrees after the holiday (*p* > 0.05) as shown in Table 6.

### 3.4. Analysis of Relaxation Styles in Male and Female Students in the Increased Pressure Group

The relaxation methods of medical students during holidays included listening to music, watching movies, exercising and chatting with friends. The results showed significant differences between males and females (*p* < 0.05) and that chatting with friends was an effective way for females to reduce levels of stress as shown in Table 7.

## 4. Discussion

During the COVID-19 pandemic, medical students may experience increased levels of stress and work-related pressures. In this study, we showed that pressure is not related to the grade levels of medical students and that female students were more stressed than their male colleagues. After a three-day holiday, the pressure on medical students showed a decreasing trend that was not statistically significant (see Table 2 and Table 4). The decrease in pressure may be related to relaxing activities such as socializing with friends, exercising, listening to music and watching movies. Amongst female medical students, chatting with friends was an effective way to reduce stress and in male medical students, exercise may be an effective way to reduce stress.

Medical students have dual identities as students and as doctors. Existing studies have shown that medical students have a higher risk of cardiovascular disease caused by stress [5]. During COVID-19, the learning mode for medical students changed from offline to online teaching [3,19,20] and students were under increased pressure when managing patients [21].

Pressure is common amongst medical students and is closely related to financial positions [22,23]. Over the past several years, the rate of growth in the economy of China has been more than 10%, but it has recently dropped to around 6%. Currently, the job market is not as strong as it had been previously due to adjustments in industrial structures which have consequently led to a lack of jobs [24]. These factors are reflected in the higher employment statistics that have been recorded in most sectors of the economy. Consequently, pursuit of higher education has become an essential requirement for medical students to ensure they remain competitive in the job market for clinical staff.

The 2019 Healthy China Action (2019–2030) report lays out specific goals and tasks for building a healthy China. In this report, it is highlighted that “strengthening mental health promotion is conducive to improving the level of public mental health and improving the public’s sense of happiness” [25,26]. Consequently, the mental health problem has received increasing attention in Chinese society.

Medical students have many common pressure problems and psychological pressures that mainly originate from the following 5 areas:

(1) A high dependency on smartphones [27,28,29]: In the digital age, the events of the world are instantly accessible on a smartphone. Although we communicate more than ever, individuals are also more isolated than ever. People need to communicate through using touch, eye contact, smell, and other connections such as laughter and other emotions.
(2) Challenges of living a double life [30]: Clinical psychologists have found that many people lead double lives. People often want to project an image of having fun with others whilst in reality they may be experiencing challenges such as overeating, overconsumption of alcohol, or experiencing periods of anxiety. Individuals are often required to work long, intense hours, resulting in persistent anxiety.
(3) Health concerns [31,32,33]: Health worries are becoming increasingly common and reflect a greater awareness of our health. For example, individuals may suspect health problems without having the appropriate health evaluations.
(4) Pressures to appear perfect [34]: In our survey, one in three of the respondents said their appearance caused them to feel anxious. We live in a world of celebrity and reality TV culture that makes ordinary people want to become idols in the public eye. People increasingly pay attention to appearance. Many people hate their appearance because comparisons between people become a source of anxiety.
(5) Young people are under increasing pressure: One survey found that the main symptoms of anxiety disorders begin at age 22 and peak at around age 32. This is in line with the eight-stage theory of psychological development proposed by the American psychologist Eric Erickson. The theory states that people in their 20′s and 30′s face the dual pressures of intimacy and loneliness [35]. People need to find the right partner, but fear that they will not be able to manage the relationship well and end up alone.

The subjects in this study reported four main methods to deal with stress as follows:

(1) Handle pressure correctly: When confronted with setbacks, people often act to shift their attention to other tasks [36] and temporarily put aside worries. For example, individuals may undertake moderate exercise and choose appropriate exercise styles according to age and endurance levels.
(2) Learn to adapt [37]: All people have individual ways of life, yet individuals should adapt to society in their own ways.
(3) Learn to release emotions [38]: When people are not satisfied, they can release unpleasant emotions through sports, entertainment, and talking to friends.
(4) Learn to communicate [37]: People should communicate more with friends as means to reduce psychological pressures.

Our study had several limitations. All of the participants were Masters degree level medical students of the China-Japan Union Hospital of Jilin University. The selected samples had regional limitations. In addition, there was no objective measurement of relaxation time and the results were all self-descriptions by the participants.

## 5. Conclusions

The COVID-19 pandemic has caused medical students to experience psychological stress. In this study, we showed that females are more stressed than males and there was a significant drop in pressure levels after holidays that may be related to relaxation activities during the holidays. For females, chatting is an effective method to reduce stress.

## Figures and Tables

**Table 1:** Results of the first PSS-14 questionnaire survey.

	High Pressure *n* = 51	Low Pressure *n* = 14	z	*p*
Total scores	31.000 (29.000–33.000)	26.000 (26.000–27.000)	−5.730	0.000
The first question	2.000 (2.000–3.000)	1.500 (0.000–2.000)	−2.958	0.003
The second question	2.000 (2.000–2.000)	1.500 (0.750–2.000)	−2.010	0.044
The third question	2.000 (2.000–3.000)	2.000 (1.750–3.000)	−1.164	0.244
The fourth question	2.000 (2.000–3.000)	2.000 (1.750–3.000)	−1.722	0.085
The fifth question	2.000 (2.000–3.000)	2.000 (1.750–2.000)	−2.402	0.016
The sixth question	2.000 (2.000–3.000)	1.860 ± 1.167	−1.746	0.081
The seventh question	2.000 (2.000–3.000)	2.000 (1.000–2.250)	−1.797	0.072
The eighth question	2.000 (1.000–2.000)	2.000 (0.750–2.000)	−0.492	0.622
The ninth question	2.000 (2.000–3.000)	2.000 (2.000–2.000)	−1.600	0.110
The tenth question	2.000 (2.000–3.000)	1.500 (0.750–2.000)	−2.213	0.027
The eleventh question	2.000 (1.000–2.000)	1.000 (0.750–2.000)	−2.034	0.042
The twelfth question	3.000 (2.000–3.000)	2.000 (2.000–3.000)	−2.205	0.027
The thirteenth question	2.000 (2.000–3.000)	2.000 (2.000–3.000)	−0.072	0.943
The fourteenth question	2.000 (2.000–3.000)	2.000 (1.000–2.000)	−2.099	0.036

Note: Questions: refer to Section 2.2 for details. No more than 2 points should be scored on a dividing line of 28. Questions with a score of ≥3 were considered to be a major source of stress. The questions in the table were as follows: (1) Upset by something happening unexpectedly? (2) Unable to control the important things in your life? (3) Nervous and stressed? (4) Dealt successfully with day-to-day problems and annoyances? (5) Effectively coping with important changes that were occurring in your life? (6) Confident about your ability to handle your personal problems? (7) Things were going your way? (8) Could not cope with all the things that you had to do? (9) Dealt successfully with irritating life hassles? (10) You were on top of things? (11) Angered because of things that were outside your control? (12) Thinking about things that you have to accomplish? (13) Able to control the way you spend your time? (14) Difficulties were piling up so high that you could not overcome them?

**Table 2:** Stratification analysis in the increased pressure group (based on the first questionnaire survey).

	Gender				Grade			
	Female (*n* = 40)	Male (*n* = 11)	z	*p*	2018 (*n* = 20)	2019 (*n* = 28)	z	*p*
Total scores	31.500 (29.000–34.00)	29.820 ± 1.888	−2.001	0.045	30.000 (29.000–35.000)	31.000 (29.000–33.000)	−0.032	0.975

Note: questions—refer to Table 1 for details.

**Table 3:** Results of the second PSS-14 questionnaire survey.

	High Pressure Group (≥28 Scores) *n* = 27	Low Pressure Group (<28 Scores) *n* = 15	z	*p*
Total scores	30.000 (29.000–34.000)	26.000 (24.000–27.000)	−5.339	0.000

Note: questions refer to Table 1 for details.

**Table 4:** Stratification analysis in the increased pressure group (Based on the second questionnaire survey).

	Gender				Grade			
	Female *n* = 22	Male *n* = 5	z	*p*	2018 *n* = 8	2019 *n* = 15	z	*p*
Total scores	31.000 (29.750–35.000)	29.000 ± 0.707	−2.302	0.021	33.750 ± 4.334	30.000 (29.000–32.000)	−1.765	0.078

Note: questions refer to Table 1 for details.

**Table 5:** Statistical analysis of the increased pressure group before and after the holiday.

	Increased Pressure Group after Holiday (≥28 Score) *n* = 27	Increased Pressure Group before Holiday (≥28 Score) *n* = 51	z	*p*
Total scores	30.000 (29.000–34.000)	31.000 (29.000–33.000)	−0.170	0.865

**Table 6:** Gender stratification in the increased pressure group before and after the holiday.

	Female				Male			
	Female group after holiday *n* = 22	Female group before holiday *n* = 40	z	*p*	male group after holiday *n* = 5	male group before holiday *n* = 11	t	*p*
Total score	31.000 (29.750–35.000)	31.500 (29.000–34.000)	−0.318	0.750	29.000 ± 0.707	29.820 ± 1.888	−0.925	0.371

**Table 7:** Relaxation methods of male and female medical students during holidays.

	Female	Male	z	*p*
	*n* = 28	*n* = 9		
music	60.000 (0.000–60.000)	35.560 ± 42.164	−0.688	0.491
exercise	0.000 (0.000–7.500)	30.000 (0.000–50.000)	−1.962	0.050
movies	0.000 (0.000–120.000)	93.330 ± 67.823	−1.591	0.112
chat	10.000 (0.000–120.000)	0.000 (0.000–0.000)	−2.075	0.038

Note: The actual number counted after the holiday was 42. Table 7 shows data from a total of 37 people, 5 of whom did not specify the specific relaxation at that time.

## Data Availability

Information on data can be obtained by contacting the first and the corresponding authors via e-mail.
